# Genome-wide identification of the NRAMP gene family in wheat and its ancestral species, and preliminary functional study of *TaNRAMP005* and *TaNRAMP006* under cadmium stress

**DOI:** 10.3389/fpls.2026.1811450

**Published:** 2026-05-07

**Authors:** Leilei Shao, Yingkun Wang, Fujing Yang, Zhiwei Sun, Zhangpeng Shi, Yilong Li, Tianzhen Lei, Yilin Li, Qin Ding, Na Niu, Lingjian Ma

**Affiliations:** College of Agronomy, Northwest Agriculture and Forestry University, Yangling, China

**Keywords:** ancestral species, Cd stress, genome-wide identification, NRAMP, *TaNRAMP005*, *TaNRAMP006*, wheat

## Abstract

Heavy metal contamination, particularly cadmium (Cd) pollution, poses a serious threat to wheat(*Triticum aestivumL.*) production and food safety. The NRAMP (Natural Resistance-Associated Macrophage Protein) gene family, which encodes metal ion transporters, plays a key role in maintaining metal homeostasis and mediating stress responses. However, systematic genome-wide analyses of the NRAMP family in wheat and its ancestral species remain limited, especially regarding studies on common wheat under Cd stress and the corresponding functional validation. This study identifies 106 NRAMP members (29 TaNRAMPs in common wheat, AABBDD genome) across wheat and its five ancestral species (AA/DD/AABB genomes), with most TaNRAMPs localized to the plasma membrane. These genes exhibited conserved yet diverse physicochemical properties. Evolutionary analysis revealed gene loss in the A, B, and D subgenomes, with segmental duplication being the main mechanism of expansion. GO and promoter analyses indicated that TaNRAMPs are involved in the transport of metal ions (such as Zn^2+^, Cd^2+^, Mn^2+^) and in responses to hormones and abiotic stresses. These genes show tissue-specific expression, and when exposed to cadmium stress, *TaNRAMP005* and *TaNRAMP006* were significantly upregulated. Moreover, heterologous expression of *TaNRAMP005* and *TaNRAMP006* in yeast markedly enhanced the yeast’s cadmium tolerance. In summary, to our knowledge, this is among the first systematic analyses of the evolutionary trajectory and functional characteristics of the NRAMP gene family in wheat and its ancestral species. Preliminary findings indicate that *TaNRAMP005* and *TaNRAMP006* play a role in the response to cadmium stress. These results provide a theoretical basis for mining genetic resources related to heavy metal tolerance and for developing strategies to improve cadmium tolerance in wheat breeding.

## 1 Introduction

Wheat (*Triticum aestivumL.*), as one of the most critical staple crops worldwide, has its safe production directly linked to global food security (Mehmood et al., 2025). However, with the escalating heavy metal pollution in agricultural ecosystems, the uptake, translocation, and tolerance mechanisms of wheat to heavy metals have emerged as a research hotspot at the intersection of agricultural and environmental sciences (Ci et al., 2010). Heavy metal pollution inhibits crop growth via seed germination disruption, photosynthesis damage, and nutrient metabolism interference (Zhao et al., 2015), while biomagnifying through the food chain to threaten human health. Therefore, elucidating the molecular mechanisms underlying plant responses to heavy metal stress holds great theoretical and practical significance (Cheng et al., 2026).

As key membrane transporters, the NRAMP (Natural Resistance-Associated Macrophage Protein) family regulates metal ion homeostasis and mediates plant responses to heavy metal stress (Thomine et al., 2003). These proteins (10–12 transmembrane domains, TMDs)​ are evolutionarily conserved, with the TMD-8 to TMD-9 region critical for divalent metal ion transport—including essential elements (e.g., Mn^2+^, Fe^2+^) and non-essential toxic metals (e.g., Cd^2+^, Pb^2+^) (Nevo and Nelson, 2006). This gene family was first identified in mouse macrophages; its encoded protein NRAMP1 participates in innate immunity against intracellular pathogens by regulating iron metabolism (Cellier et al., 1995), while its homologous protein NRAMP2 (DMT1) is widely involved in the absorption and homeostasis maintenance of multiple divalent metal ions (Fe^2+^, Mn^2+^, Zn^2+^, Cu^2+^, Cd^2+^, Pb^2+^) in vertebrates (Gunshin et al., 1997).

With the rapid development of genomics and bioinformatics, significant progress has been made in researching the plant NRAMP gene family. Six NRAMP members have been identified in Arabidopsis thaliana, which were divided into two subfamilies via phylogenetic analysis. The functions of five members (*AtNRAMP1–AtNRAMP5*) have been experimentally validated, revealing their roles in metal-specific translocation (e.g., iron, manganese) (Arif et al., 2025). The rice (Oryza sativa) genome contains seven NRAMP genes, most of which have been confirmed to be involved in heavy metal stress responses (Ishimaru et al., 2012). Thirteen NRAMP genes identified in soybean (Glycine max) provide important genetic resources for studying heavy metal tolerance mechanisms in leguminous plants (Qin et al., 2017).

Notably, Cd transport functions have been extensively documented (Seregin and Ivanov, 2001). For example, *OsNRAMP5* can reduce cadmium accumulation in grains. Meanwhile, the *OsNRAMP5* mutant rice increases the deposition of cadmium in the cellulose of root cell walls and reduces the transport of cadmium in the xylem and phloem of the plant (Zhang et al., 2024). *AtNRAMP6* increased the sensitivity of yeast cells to Cd, and Arabidopsis overexpressing *AtNRAMP6* showed enhanced cadmium hypersensitivity (Cailliatte et al., 2009). Knocking out *TaNRAMP5* can effectively reduce cadmium (Cd) accumulation in wheat (Cheng et al., 2025). However, there remains a gap in the systematic identification and functional analysis of this gene family in wheat and its ancestral species. In this study, we comprehensively screened and identified NRAMP gene family members in wheat and its ancestors using bioinformatic approaches, and analyzed their evolutionary origins and phylogenetic relationships via comparative genomics. Additionally, we performed functional annotation of wheat NRAMP genes from multiple dimensions (gene structure, conserved motif analysis, promoter cis-element composition), combined with expression profile data to dissect their spatiotemporal expression patterns, and verified the responses of NRAMP members to cadmium stress using real-time quantitative PCR. This work lays a theoretical foundation for revealing the functional mechanisms of the NRAMP gene family in wheat and its ancestors and mining their stress resistance genetic resources. It also provides research directions for subsequent studies on molecular cadmium translocation and its underlying mechanisms.

## 2 Materials and methods

### 2.1 Identification and preliminary analysis of NRAMP genes in wheat (*Triticum aestivumL.*)

Obtain the whole-genome data of wheat and its ancestral species from the Ensembl Plants database (https://plants.ensembl.org/index.html). Simultaneously, download the protein sequences of known NRAMP family members from *Arabidopsis thaliana* and *Oryza sativa* from the Pfam database (https://pfam.xfam.org/). Use the HMMER3.0 program to analyze the whole genomes of wheat and its ancestral species (*Aegilops tauschii*, *Triticum turgidum*, *Triticum urartu*, *Triticum dicoccoides*, and *Triticum spelta*) with a screening threshold of e < 1e-5 to identify proteins containing the Nramp domain (PF01566).

At the same time, employ the BLASTP program, using the protein sequences of known NRAMP members in rice and Arabidopsis as query sequences, to perform a homology search against the protein dataset. This BLASTP search also adopts an e-value cutoff of <1e-5 and a protein identity threshold of 50% as selection criteria. Integrate the protein information obtained from both HMMER3.0 and BLASTP analyses to preliminarily predict the NRAMP genes in wheat and its ancestral species. To confirm these preliminary predictions, the NCBI-CDD web server (https://www.ncbi.nlm.nih.gov/Structure/cdd/wrpsb.cgi) is ultimately used to validate the candidate NRAMP genes in wheat.

After obtaining the candidate NRAMP genes in wheat, to further investigate their characteristics, the ExPASy server (https://web.expasy.org/compute_pi/) is utilized to predict the theoretical isoelectric point (pI) and molecular weight (MW) of TaNRAMP proteins. Meanwhile, the CELLO web server (http://cello.life.nctu.edu.tw/) is employed to predict their subcellular localization.

### 2.2 Phylogenetic analysis

A phylogenetic analysis was conducted on the NRAMP protein sequences of Arabidopsis thaliana, rice, wheat, and their ancestral species. First, the NRAMP protein sequences were imported into MEGA 6.0 software and aligned using the ClustalW algorithm with parameters set as follows: Gap Opening Penalty = 10.00 and Gap Extension Penalty = 0.10, to obtain high-quality sequence alignment. Subsequently, the “Neighbor-Joining” (NJ) method was selected in MEGA 6.0 to construct the phylogenetic tree. Genetic distances were calculated based on the alignment results using the default p-distance model, which is suitable for highly similar sequences among closely related species, and 1,000 bootstrap replicates were performed to evaluate nodal support. Based on the above analysis, MEGA 6.0 generated an unrooted Neighbor-Joining (NJ) phylogenetic tree file, and the final tree was visualized with EvolView (https://www.evolgenius.info/evolview/#/treeview).

### 2.3 Chromosomal localization, gene structure, and conserved motif analysis of TaNRAMPs

The chromosomal locations of TaNRAMP genes were extracted from the wheat genome annotation file in Gff3 format and visualized on wheat chromosomes. This study conducted an in-depth analysis of the gene structures and coding sequences (CDS) of TaNRAMP genes, exploring the distribution characteristics of exons. The MEME tool (https://meme-suite.org/meme/doc/meme.html) was employed to identify conserved motifs in TaNRAMP proteins, with the optimal motif size set between 10 and 200 amino acids and a maximum of 12 motifs allowed. Finally, the phylogenetic tree, gene structures, and conserved motifs of wheat NRAMP genes were integrated and visualized on the EvolView platform (https://www.evolgenius.info/evolview-v2/#mytrees/2/2).

### 2.4 Syntenic analysis between wheat and its five ancestral species

Collinearity analysis of TaNRAMPgenes in wheat and its five ancestral species (Aegilops tauschii, Triticum urartu, Triticum dicoccoides, Triticum turgidum, and Triticum spelta) was performed using MCScanX. The duplication events of TaNRAMPgenes and their collinear relationships with other species were visualized using Circos (v0.67). Coding DNA sequences (CDS) and protein sequences of collinear gene pairs were aligned to calculate Ka/Ks ratios using TBtools. Finally, the divergence times of collinear gene pairs were estimated using the formula T=Ks/(2 λ× 10−6) Mya, where λ= 6.5 × 10 − 9.

### 2.5 GO annotation of TaNRAMP

The GO annotations of wheat NRAMP proteins were obtained from the Plaze database (https://www.vandepoelelab.be/plaza/versions/plaza_v3_dicots/) and the Plant Transcriptional Regulation Map Database (http://plantregmap.gao-lab.org/). The GO annotation results were visualized using the online tool WEGO (http://wego.genomics.org.cn/).

### 2.6 Promoter analysis of TaNRAMP genes

The upstream 2 kb DNA sequences of TaNRAMP genes were downloaded from the Ensembl Plant database, and cis-regulatory elements in the promoter regions were predicted using the PLACE database (bioinformatics.psb.ugent.be/webtools/plantcare/html/).

### 2.7 Gene expression and qRT-PCR analysis of TaNRAMPs

To gain in-depth insights into the tissue-specific expression and stress response patterns of TaNRAMPs, the expression profiles of TaNRAMP genes in roots, stems, leaves, and spikes of Chinese Spring wheat, as well as their responses to various stress conditions, were downloaded from the Wheatomics website. The expression data were subsequently visualized using GraphPad Prism 8 software.

To investigate the response of TaNRAMP genes to cadmium (Cd) stress, a hydroponic experiment was conducted on October 3, 2024, using the wheat cultivar Fielder. Selected plump Fielder wheat seeds​ were surface-sterilized with 1% H_2_O_2_ for 12 h and germinated on filter paper in Petri dishes for 3 days. Subsequently, uniformly germinated seedlings were transplanted into hydroponic boxes. The plants were cultivated under the following conditions: a temperature of 22 °C, a 16/8 h light/dark photoperiod, and a light intensity of 10,000 lux. The control group was treated with standard Hoagland nutrient solution, while the treatment group received Hoagland solution supplemented with 200 μM Cd. Each group contained 10 biological replicates (plants). The nutrient solution was renewed every 3 days. After 14 days of continuous treatment, phenotypic photographs were taken of three randomly selected plants from each group. Root and leaf samples were then collected and immediately stored at -80°C for subsequent analysis. Total RNA was extracted from the Root samples using RNAiso reagent, and cDNA was synthesized using the RT Master Mix Perfect Real Time Kit (Takara Bio, Dalian, China). To detect the expression of the TaNRAMP genes, specific primers were designed based on the NCBI database(Additional file: **Supplementary Table 2**). Quantitative real-time reverse transcription polymerase chain reaction (qPCR) was performed to analyze the expression of TaNRAMP genes using QuantStudio Real-Time PCR software. The relative expression levels of 29 TaNRAMP genes were calculated using the 2^(−ΔΔCt) method. The actin gene *TraesCS6B02G243700* was selected as the internal reference gene for normalizing the expression levels of TaNRAMP genes.

### 2.8 Yeast complementation assay of *TaNRAMP005* and *TaNRAMP006* genes

To investigate the cadmium (Cd) tolerance function of *TaNRAMP005* and *TaNRAMP006* in yeast, the full-length coding sequences (CDS) of these two genes were respectively cloned into the yeast expression vector pYES3, which had been digested with BamHI, vector pYES3 was preserved and provided by our laboratory. The specific primers used for amplification are listed in Additional file: **Supplementary Table 10**. Accordingly, the recombinant expression plasmids pYES3-*TaNRAMP005* and pYES3-*TaNRAMP006* were successfully constructed. Subsequently, the empty vector pYES3 and its recombinant vectors were separately transformed into competent cells of the cadmium-sensitive yeast mutant strain Δycf1(The Δycf1 strain was constructed from the BY4741 yeast strain via endogenous knockout of the YCF1​ gene. YCF1 (Yeast Cadmium Factor 1) mediates the chelation of heavy metals with glutathione and their subsequent vacuolar sequestration to alleviate cellular stress responses; mutation of this gene results in increased yeast sensitivity to cadmium.). Three yeast strains were initially inoculated into SD-Trp (synthetic glucose medium lacking tryptophan) liquid medium and cultured until the OD_600_ reached 1.5. Subsequently, the cells were harvested by centrifugation and the supernatant was discarded. The pellets were resuspended in SC-Trp (synthetic complete medium lacking tryptophan but devoid of sugar) and shaken for 1 h to eliminate glucose interference. Serial dilutions (1, 10^−1^, 10^−2^, 10^−3^, 10^−4^, and 10^−5^) were then performed sequentially. Next, 5 μL aliquots of each dilution were spotted onto SG-Trp (synthetic galactose medium lacking tryptophan) agar plates supplemented with different concentrations of Cd (0, 30, 40, and 50 μM) to initiate induction. The plates were incubated at 30°C for 48 h, after which the growth phenotypes of the strains under varying Cd concentrations were observed and recorded. To further evaluate the growth of yeast under cadmium (Cd) stress, this study performed shake flask cultures using SG-Trp liquid medium supplemented with 0 and 30 μM Cd for both empty pYES3-transformed yeast and *TaNRAMP005/006*-overexpressing yeast. Samples were collected at seven time points: 0, 12, 24, 36, 48, 60, and 72 hours. The optical density of the culture (OD_600_) was measured at a wavelength of 600 nm using a UV-Vis spectrophotometer to monitor changes in the growth curve.

## 3 Results

### 3.1 Genome-wide identification and physicochemical property analysis of wheat NRAMP genes

Through genome-wide screening and identification in common wheat and its ancestral species (*Triticum urartu, Aegilops tauschii, Triticum turgidum, Triticum dicoccoides, and Triticum spelta*), NRAMP family members were preliminarily identified using both HMMER’s hmmsearch and NCBI’s Blastp methods. Domain prediction via CD-search was further employed for validation. A total of 11 *Triticum urartu* NRAMP genes (TuNRAMP, AA genome), 12 *Aegilops tauschii* (AeNRAMP, DD genome), 23 *Triticum turgidum* (TtNRAMP, AABB genome), 22 *Triticum dicoccoides* genes (TdNRAMP, AABB genome), 29 *Triticum spelta* genes (TsNRAMP, AABBDD genome), and 29 common wheat genes (TaNRAMP, AABBDD genome) were ultimately identified. Analysis of the physicochemical properties of the identified members revealed that the smallest protein consisted of 83 aa in *Triticum dicoccoides*, while the longest protein (1240 aa) was found in both common wheat and *Triticum spelta.* The isoelectric point (pI) ranged from 4.67 to 9.77, indicating a broad charge distribution from acidic to alkaline among family members. Subcellular localization predictions showed that most NRAMP genes are located on the plasma membrane, though a few were predicted to localize to the nucleus, vacuolar membrane, extracellular space, and chloroplast. Further analysis of the common wheat TaNRAMP family showed that their pI values ranged from 4.99 (TaNRAMP007, TaNRAMP010, TaNRAMP014) to 8.63 (TaNRAMP025), with relative molecular weights (MW) between 53,001.19 (TaNRAMP019) and 135,275.04 (TaNRAMP023), corresponding to protein lengths of 487 aa (TaNRAMP019) to 1240 aa (TaNRAMP023). Subcellular localization predictions via the Cello website indicated that all TaNRAMPs are located on the plasma membrane.

### 3.2 Phylogenetic analysis

**Figure 1:**
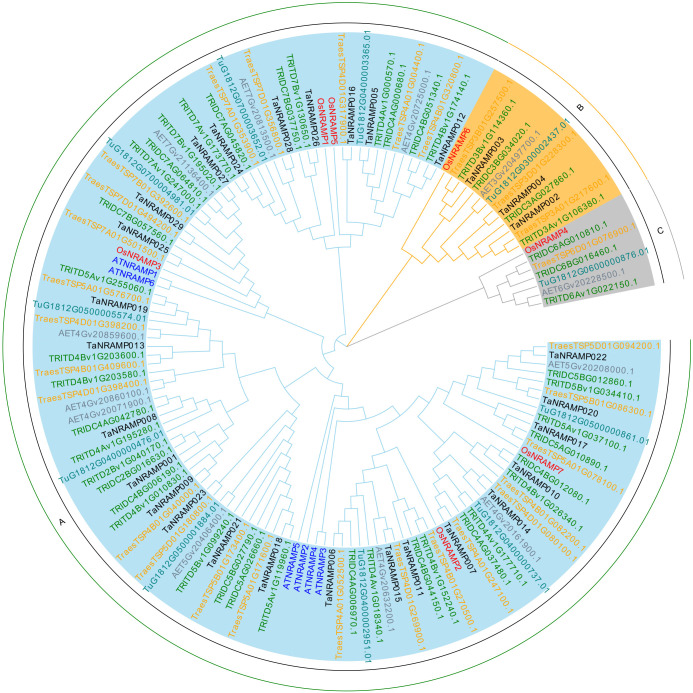
Phylogenetic tree of the *NRAMP* gene family. This phylogenetic tree was primarily constructed using *NRAMP* gene family members from rice, Arabidopsis, Triticum urartu, Aegilops tauschii, Triticum turgidum, Triticum dicoccoides, Triticum spelta, and common wheat. **(A–C)** indicate the three subgroups of the gene family.

To investigate the phylogenetic relationships between common wheat and its ancestral species, this study identified NRAMP genes in the ancestors of wheat (**Figure 1**) *(Triticum urartu, Aegilops tauschii, Triticum turgidum, Triticum dicoccoides, and Triticum spelta*). A total of 11 TuNRAMP genes were identified in *Triticum urartu* (AA genome), 12 AeNRAMP genes in *Aegilops tauschii* (DD genome), 23 TtNRAMP genes in *Triticum turgidum* (AABB genome), 22 TdNRAMP genes in *Triticum dicoccoides* (AABB genome), and 29 TsNRAMP genes in *Triticum spelta* (AABBDD genome). Further analysis of the distribution of NRAMP genes across the subgenomes of common wheat (AABBDD genome) revealed that subgenome A contains 10 genes, subgenome B contains 11 genes, and subgenome D contains 8 genes. Comparative analysis with the number of NRAMP genes in ancestral species indicated that, during the evolution from ancestral species (such as the AABBDD genomes of *Triticum dicoccoides*/*Triticum spelta*) to common wheat, varying degrees of gene loss occurred in the NRAMP genes across the A, B, and D subgenomes.

### 3.3 Collinearity analysis

Gene family members are typically generated through gene duplication events (such as whole-genome duplication or tandem duplication). Analyzing the collinearity of gene families across different species can help infer the mechanisms behind their duplication (e.g., single vs. multiple duplications, local vs. whole-genome duplications), as well as reveal the sequence of duplication events and their evolutionary trajectories. Therefore, we analyzed gene duplication events within the TaNRAMP gene family, calculated Ka/Ks ratios, and estimated divergence times (Additional file: **Supplementary Table 3**).

A total of 29 duplicated pairs were detected among TaNRAMP genes in wheat. With the exception of *TaNRAMP001* and *TaNRAMP020*, all other TaNRAMP members were involved in duplication events, indicating that segmental duplication has been the primary mechanism for the expansion of the TaNRAMP family in wheat. The presence of multiple homologous genes further confirms the evolutionary conservation of this gene family. The Ka/Ks ratios in wheat were all below 1, suggesting that NRAMP genes have undergone purifying selection in wheat varieties. Divergence time estimates ranged from 0.8 to 123 million years ago (mya), revealing that the TaNRAMP gene family has not only experienced purifying selection throughout wheat evolution but also functional divergence, with some genes retaining functional redundancy after multiple duplication events.

**Figure 2:**
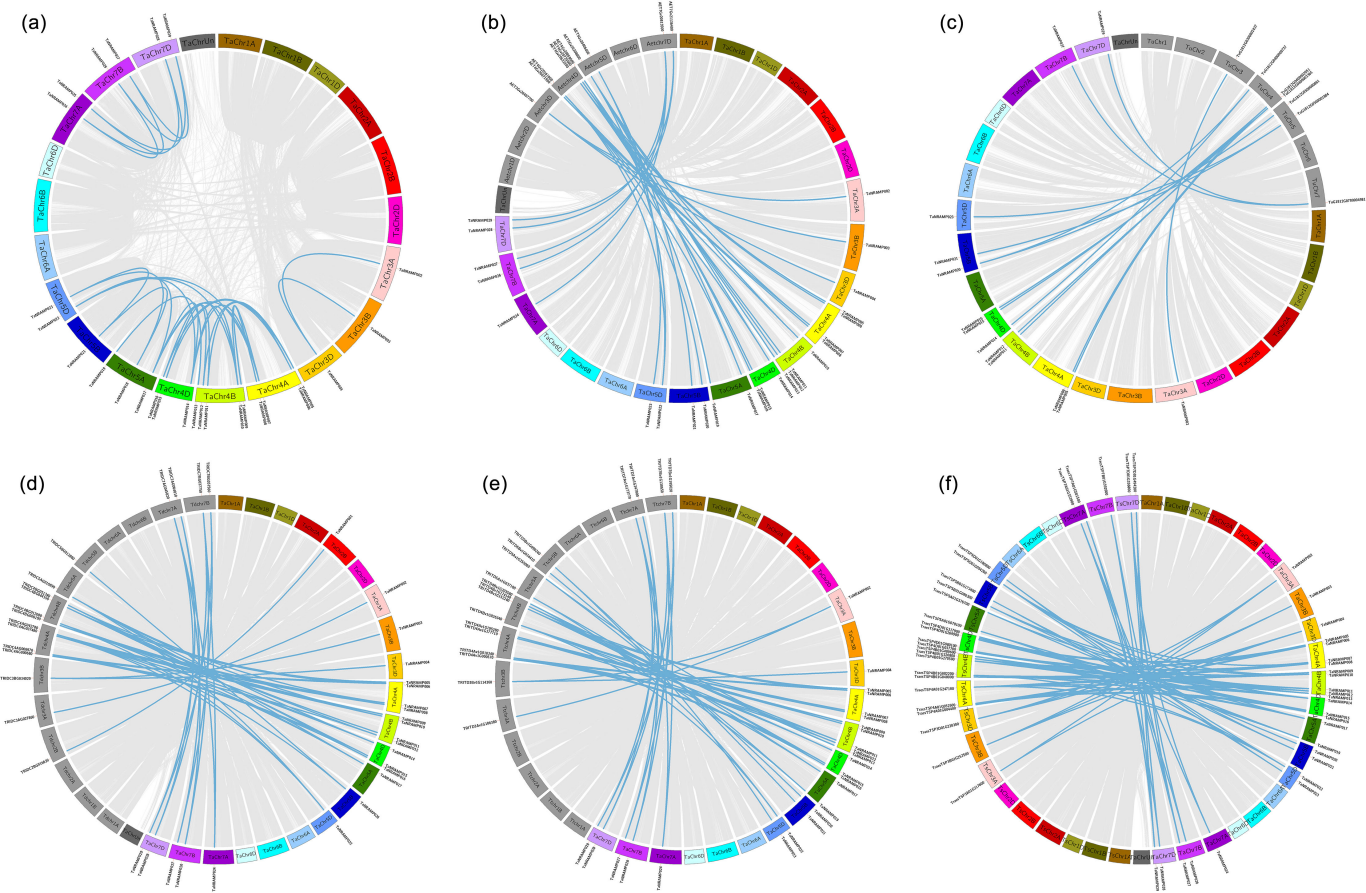
Synteny analysis of *NRAMP* genes. **(a)** Duplicate gene pairs in the wheat genome; synteny analysis of *TaNRAMP* with **(b)** *Aegilops tauschii*, **(c)** Triticum urartu, **(d)** *Triticum dicoccoides*, **(e)** *Triticum turgidum*, and **(f)** *Triticum spelta*. Thin lines represent syntenic blocks across the whole genomes of the four species, while dark lines indicate syntenic gene pairs of *NRAMP*.

To understand the origin and evolutionary relationships of the NRAMP gene family, genome-wide collinearity analysis was conducted between wheat and its ancestral species (*Aegilops tauschii, Triticum urartu, Triticum dicoccoides, Triticum turgidum*, and *Triticum spelta*). The collinearity results showed 29 duplicated gene pairs within wheat itself, while 30, 14, 46, 44, and 74 collinear gene pairs were identified between wheat and *Aegilops tauschii, Triticum urartu, Triticum dicoccoides, Triticum turgidum*, and *Triticum spelta*, respectively (**Figure 2**).

In some collinear gene pairs, neither synonymous (Ks) nor nonsynonymous (Ka) substitutions were detected, likely due to the close phylogenetic relationship between wheat and its ancestors, which limited sequence divergence. As a result, divergence time could not be calculated for these pairs. For the remaining collinear pairs, all Ka/Ks values were less than 1, indicating that NRAMP genes have undergone purifying selection during the evolution of wheat and these ancestral species.

The divergence times between wheat and its related species ranged widely. Between wheat and *Aegilops tauschii* (Additional file: **Supplementary Table 4**), the longest was about 108 mya, with an average of 25 mya; between wheat and *Triticum urartu* (Additional file: **Supplementary Table 5**), the longest was about 110 mya, with an average of 13 mya; between wheat and *Triticum dicoccoides* (Additional file: **Supplementary Table 6**), the longest was around 128 mya, with an average of 25 mya; and between wheat and *Triticum turgidum* (Additional file: **Supplementary Table 7**), the longest was approximately 115 mya, with an average of 13 mya; The longest divergence time between wheat and *T. spelta* was approximately 123 mya, with an average of 23 mya(Additional file: **Supplementary Table 8**);

Overall, these results demonstrate that the wheat NRAMP gene family shares a close phylogenetic relationship with its ancestral species (*Aegilops tauschii, Triticum urartu, Triticum dicoccoides, Triticum turgidum, and Triticum spelta*) and has maintained functional conservation throughout long-term evolution.

### 3.4 Chromosomal localization, gene structure, and conserved motif analysis of TaNRAMPs

**Figure 3:**
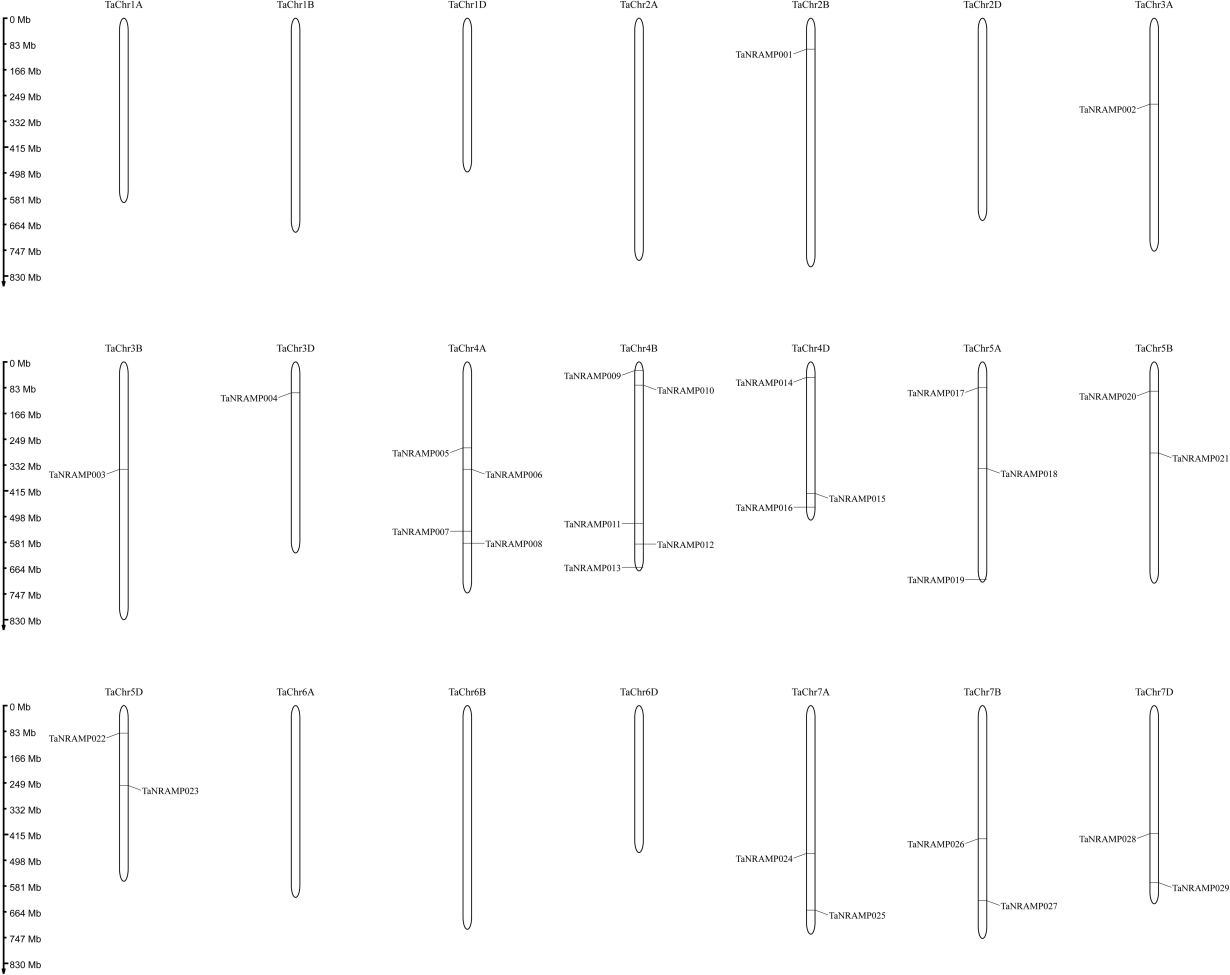
Chromosomal localization of *TaNRAMP* genes.

Analysis of chromosomal localization information obtained from the wheat genome annotation file (Gff3) revealed that only subgenome B of chromosome 2 contains one TaNRAMP gene, while chromosomes 3, 4, 5, and 7 harbor varying numbers of TaNRAMP genes across all three subgenomes (A, B, and D) (**Figure 3**). Further examination and visual mapping of the exon–intron structures of the TaNRAMP gene family showed significant variation in exon numbers (ranging from 4 to 13), with some genes lacking UTR regions. Phylogenetic analysis indicated that TaNRAMP genes within the same evolutionary branch exhibit high similarity in exon and intron numbers, while clear differences were observed across different branches. This pattern highlights both structural conservation and considerable diversity within the TaNRAMP gene family during evolution.

**Figure 4:**
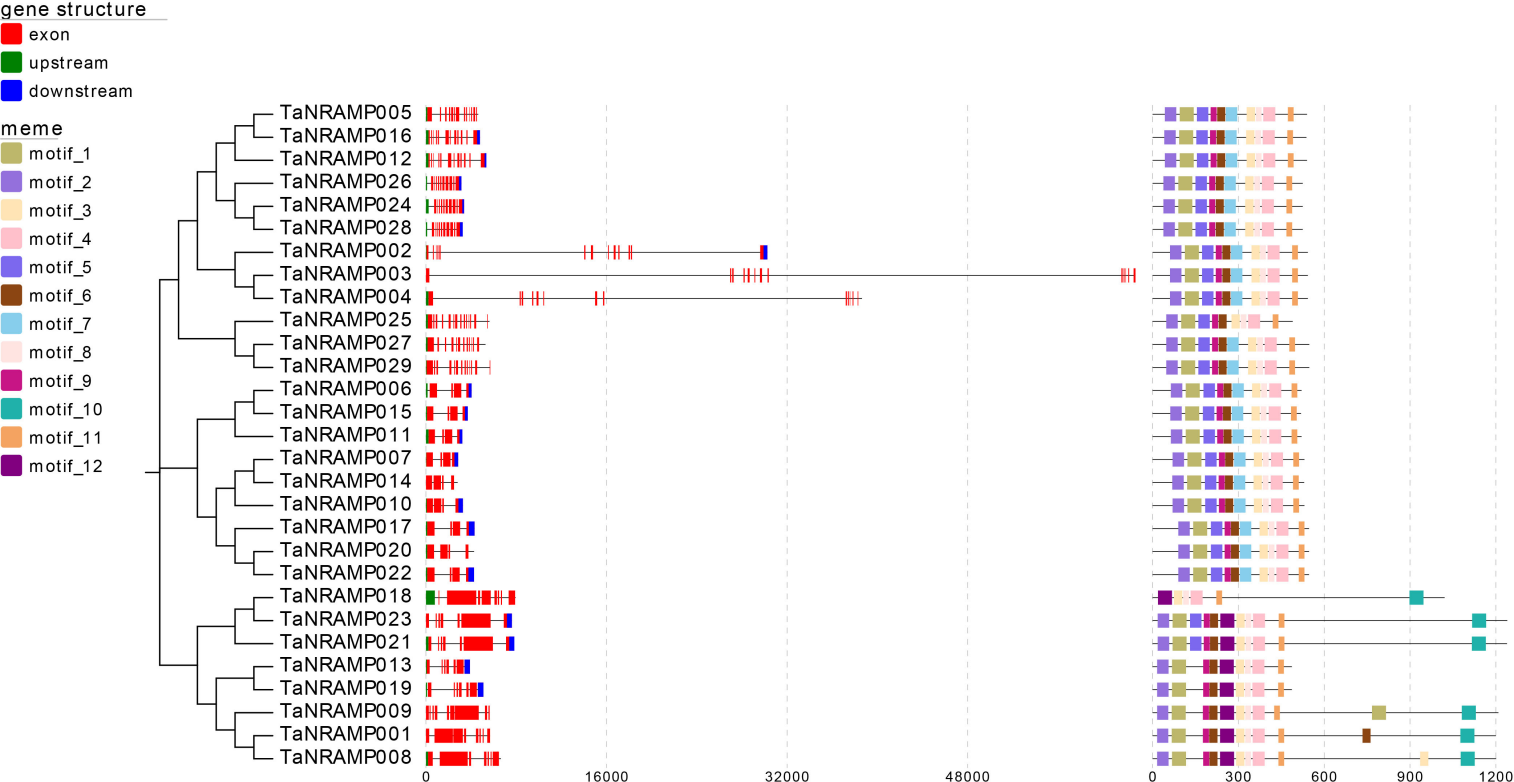
Gene structure and conserved motif analysis of *TaNRAMPs* based on the phylogenetic tree. Exons are represented by red boxes, the 5' untranslated region (5' UTR) by green boxes, and the 3' untranslated region (3' UTR) by blue boxes. Different motifs are represented by colored boxes. The gene structure corresponds to the length of the gene sequence at the bottom, while the conserved motifs correspond to the length of the protein sequence at the bottom.

To investigate sequence similarity among TaNRAMP genes and predict their potential functions, the sequences of all 29 TaNRAMP genes were submitted to the MEME online platform for conserved motif analysis (**Figure 4**). The results demonstrated that members of the TaNRAMP gene family generally share similar motif architectures, though notable differences exist across evolutionary branches. For example, TaNRAMP018 lacks several conserved motifs at its front end—including motif_2, motif_1, motif_5, motif_9, and motif_6—compared to other members within the same branch. In a highly similar branch consisting of TaNRAMP023, TaNRAMP021, TaNRAMP013, TaNRAMP019, TaNRAMP001, TaNRAMP008, and TaNRAMP009, motifs 5 and 7 are generally absent, but motif_12 is uniquely present. Furthermore, TaNRAMP023 and TaNRAMP021 specifically lack motif_7 but possess motif_10, unlike other branches. Most other branches show high internal structural consistency, with the exception of TaNRAMP025, which uniquely lacks motif_7.

### 3.5 GO annotation and promoter analysis of TaNRAMP

**Figure 5:**
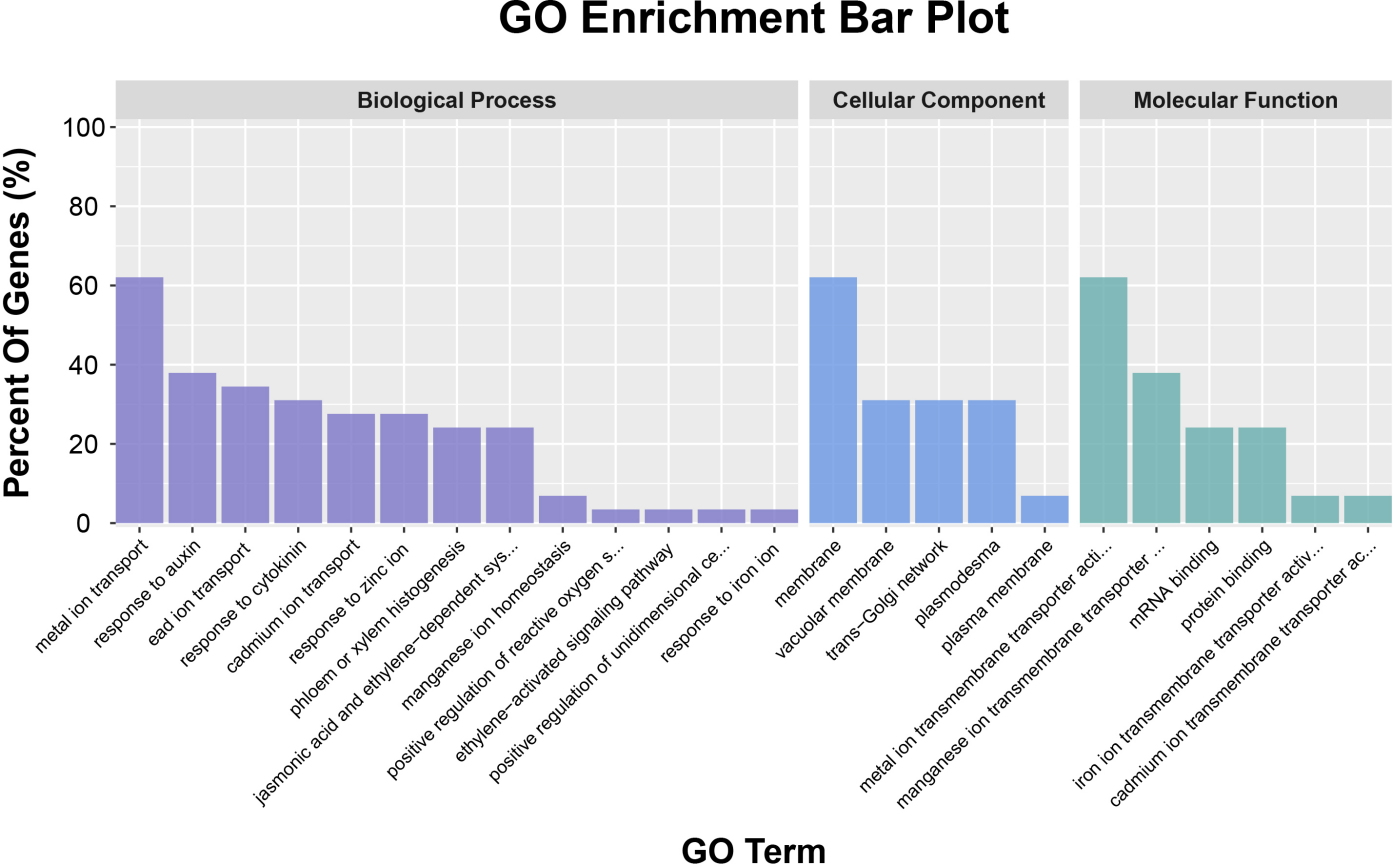
GO annotation results of TaNRAMP proteins. The x-axis represents the types of GO annotations, mainly divided into three categories: cellular component, molecular function, and biological process; the y-axis represents the proportion of genes in each GO category.

Based on GO annotation, an analysis of the 29 members of the wheat NRAMP gene family was conducted to predict the potential biological processes, cellular components, and molecular functions in which TaNRAMP members might be involved (**Figure 5**; Additional file: **Supplementary Table 9**). In terms of biological processes, 18 TaNRAMP genes were enriched in metal ion transport, eight were simultaneously enriched in response to zinc ion, lead ion transport, and cadmium ion transport, two were involved in manganese ion homeostasis, and one was primarily associated with response to iron ion. Additionally, some members exhibited responsiveness to hormone treatments, mainly enriched in response to auxin and response to cytokinin. Several TaNRAMP genes were also implicated in processes such as phloem or xylem histogenesis, jasmonic acid and ethylene-dependent systemic resistance, and the ethylene-mediated signaling pathway.

In molecular function, 18 TaNRAMP genes were enriched in metal ion transmembrane transporter activity, and 11 members were enriched in manganese ion transmembrane transporter activity. Regarding cellular component, 18 TaNRAMP members were enriched in membrane localization, nine were simultaneously enriched in vacuolar membrane, trans-Golgi network details, and plasmodesma, while two were enriched in the plasma membrane. These findings suggest that TaNRAMPs primarily function in metal ion transport and related regulatory processes within membrane systems, particularly at key transport sites such as the plasma membrane and vacuolar membrane.

**Figure 6:**
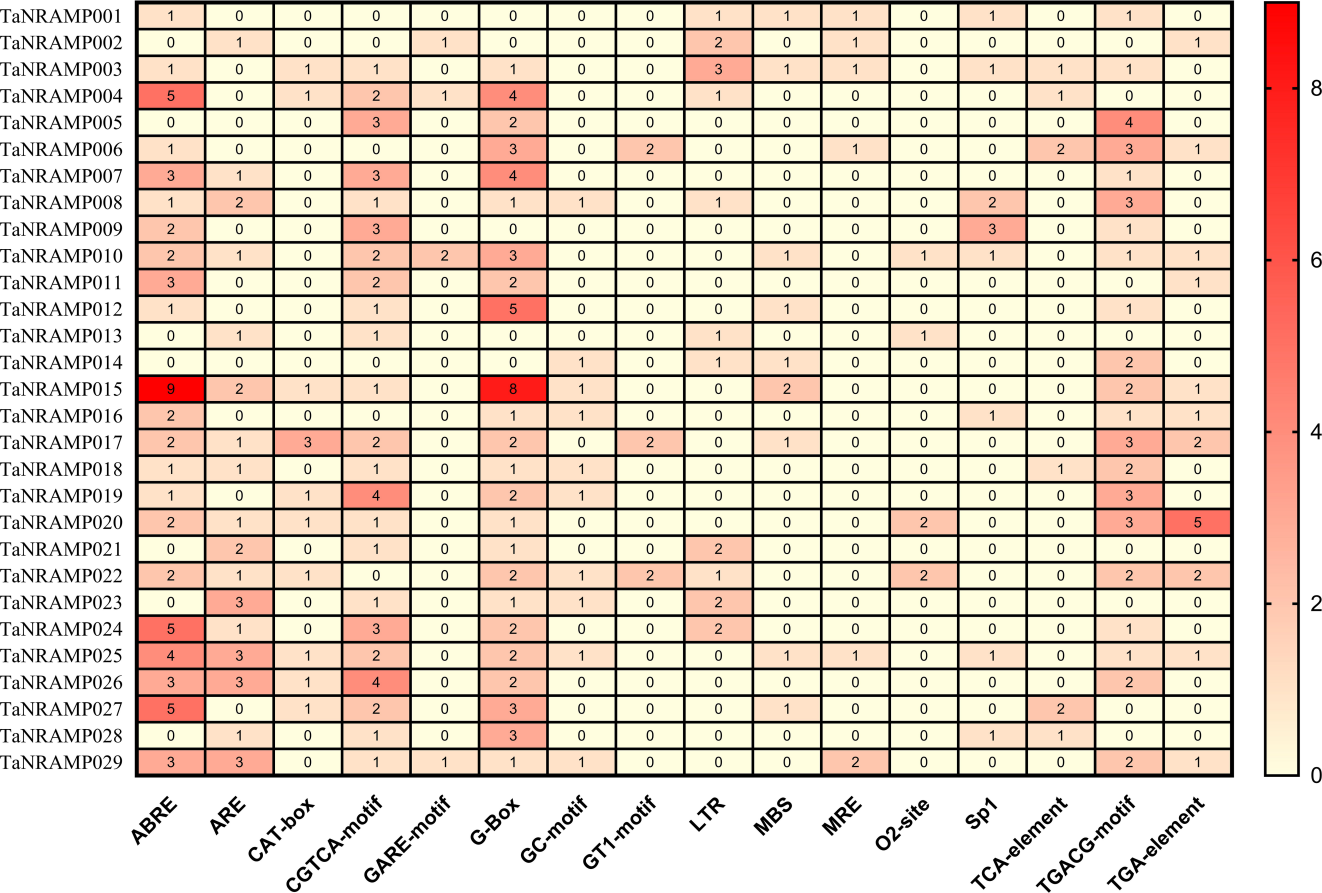
Promoter analysis of *TaNRAMP* genes. Cis-acting elements in the promoters of 29 TaNRAMP genes, with highly enriched elements shown in red and less enriched elements shown in yellow.

Gene expression is regulated by cis-acting elements, which are generally located in the promoter regions of genes. Therefore, we predicted potential cis-acting elements within the 2 kb upstream promoter sequences of the 29 TaNRAMP genes. The results revealed that the promoter regions of the TaNRAMP gene family are widely enriched with various cis-elements related to hormones (ABA, JA, SA), abiotic stresses (drought, low temperature, heavy metals), and developmental regulation (**Figure 6**).

### 3.6 Gene expression and qRT-PCR analysis of TaNRAMPs

**Figure 7:**
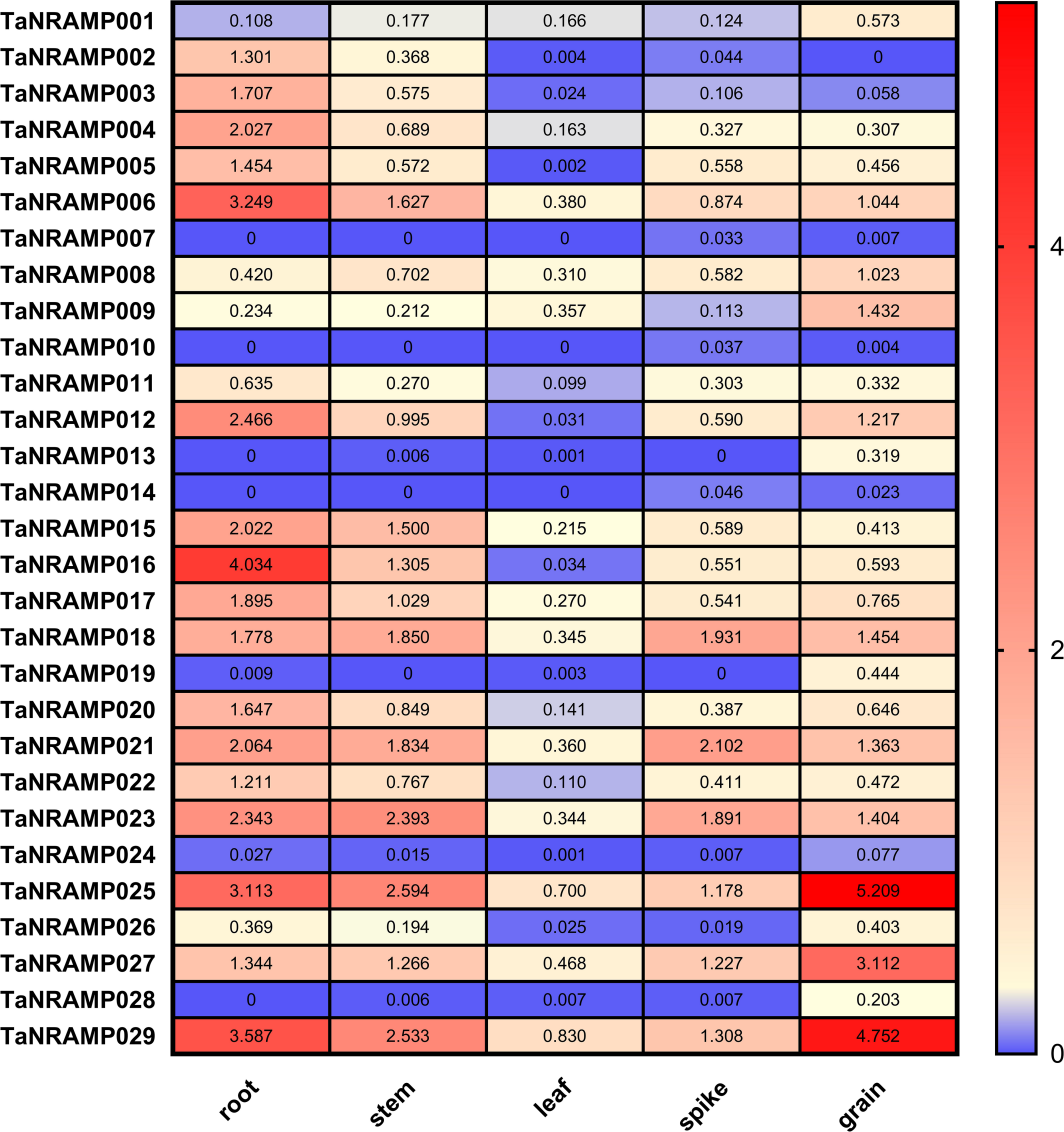
Expression patterns of *TaNRAMP* genes. Expression profiles of 29 *TaNRAMP* genes in roots, stems, leaves, and spikes. High expression levels are shown in red, and low expression levels are shown in blue.

Expression data of TaNRAMPs in roots, stems, leaves, and spikes of Chinese Spring were downloaded from the Wheatomics website (**Figure 7**). The results showed that most genes, including *TaNRAMP006*, *TaNRAMP011*, *TaNRAMP015*, *TaNRAMP017*, *TaNRAMP018*, *TaNRAMP020*, *TaNRAMP021*, *TaNRAMP022*, and *TaNRAMP023*, were highly expressed in roots. Some TaNRAMP genes, such as *TaNRAMP001*, *TaNRAMP008*, *TaNRAMP009*, *TaNRAMP013*, *TaNRAMP019*, *TaNRAMP024*, *TaNRAMP025*, *TaNRAMP026*, *TaNRAMP027*, *TaNRAMP028*, and *TaNRAMP029*, exhibited higher expression in grains. Additionally, *TaNRAMP007*, *TaNRAMP010*, and *TaNRAMP014* showed relatively high expression in spikes. These findings indicate that TaNRAMP genes exhibit tissue-specific expression patterns.

**Figure 8:**
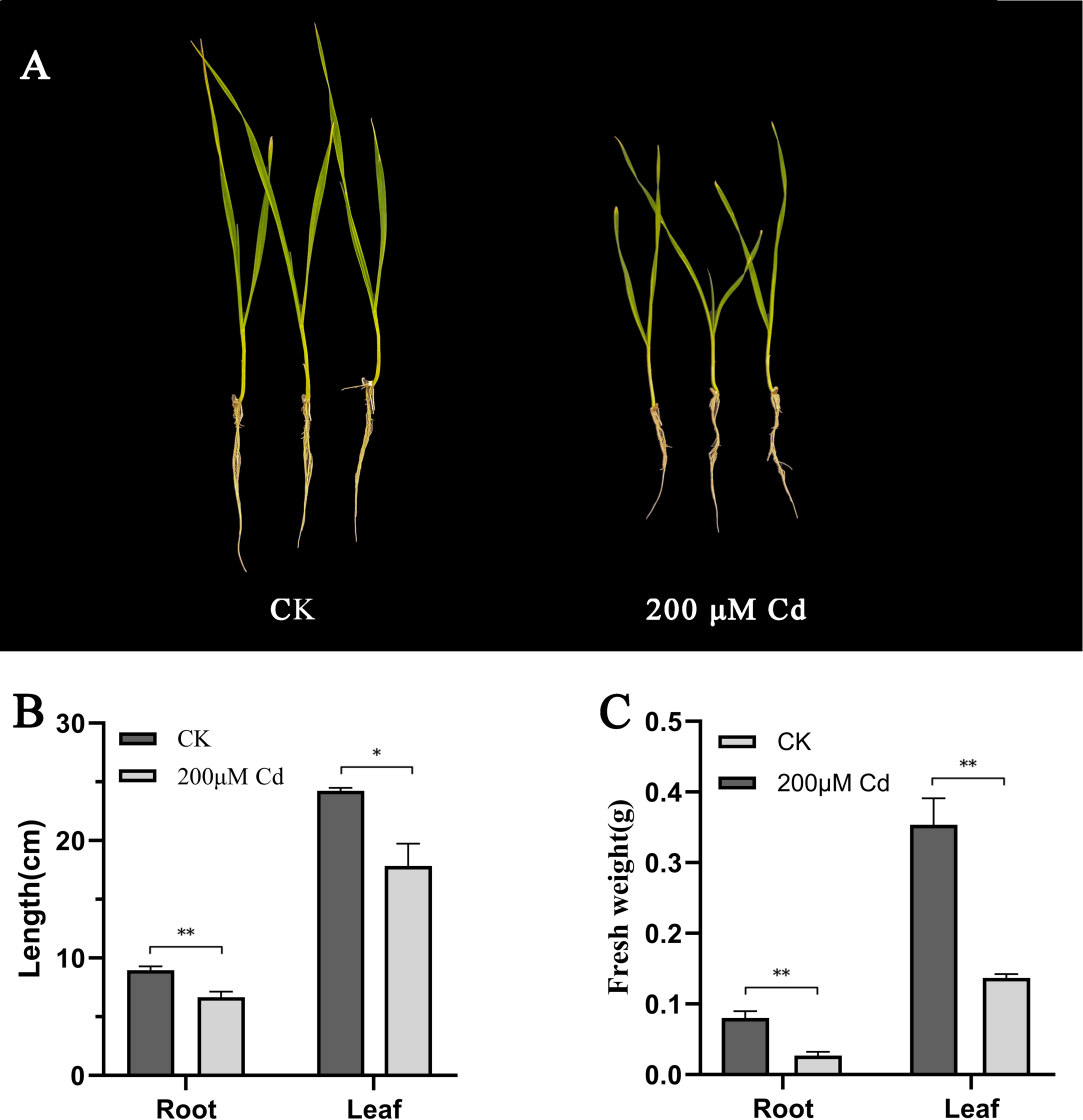
Growth phenotypes and physiological parameters of Fielder wheat under cadmium stress. **(A)** Representative images of Fielder wheat plants after 14-day exposure to 0 or 200 µmol/L Cd^2+^. **(B)** Root length and leaf length. **(C)** Fresh weight of roots and leaves. (three biological replicates); data are means ± SD, with significance determined by ANOVA and Tukey’s test after checking assumptions (*P < 0.05, **P < 0.01).

**Figure 9:**
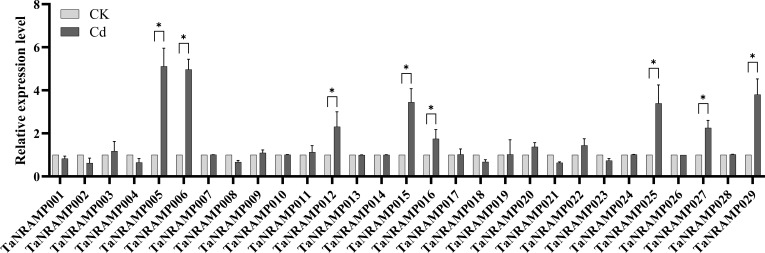
Quantitative reverse transcription-polymerase chain reaction (qRT-PCR) analysis of *TaNRAMP* genes. Relative expression levels of 29 genes under 200 μM cadmium treatment. (three biological and three technical replicates); data are means ± SD, with significance determined by ANOVA and Tukey’s test after checking assumptions (P < 0.05).

During the seedling stage, hydroponic treatment with CdCl_2_ was applied to the wheat cultivar Fielder at two concentrations: 0 μM and 200 μM. After 14 days of stress treatment, the Fielder cultivar exhibited significant growth inhibition under 200 μM Cd exposure, specifically characterized by suppressed elongation of both roots and shoots, along with a marked reduction in overall plant biomass (**Figure 8**). Root and leaf samples were subsequently collected and stored at -80°C. Concurrently, total RNA was extracted from the samples, and the relative expression levels of the TaNRAMP family genes were analyzed using quantitative real-time PCR (qRT-PCR) technology (**Figure 9**; **Supplementary Table 2**). The results revealed diverse responses among different TaNRAMP genes under cadmium stress. *TaNRAMP001*, *TaNRAMP002*, *TaNRAMP004*, *TaNRAMP008*, *TaNRAMP018*, *TaNRAMP021*, and *TaNRAMP023* were significantly downregulated. Phylogenetic analysis indicated that *TaNRAMP001*, *TaNRAMP008*, and *TaNRAMP009* are homologous copies derived from different subgenomes, and a similar relationship was observed among *TaNRAMP002*, *TaNRAMP003*, *TaNRAMP004*, *TaNRAMP018*, *TaNRAMP021*, and *TaNRAMP023*. Genes within the same phylogenetic branch generally showed similar expression trends under cadmium stress; however, functional complementarity was also observed. For example, *TaNRAMP003* and *TaNRAMP009* did not respond noticeably when their homologs were downregulated.

Some TaNRAMP genes were upregulated, including *TaNRAMP005*, *TaNRAMP012*, and *TaNRAMP016*, which belong to the same branch and were all induced. Similarly, *TaNRAMP006* and *TaNRAMP015* in another branch were simultaneously upregulated, as were *TaNRAMP025*, *TaNRAMP027*, and *TaNRAMP029*. These results suggest that different phylogenetic branches of TaNRAMP genes respond differently to cadmium stress, while members within the same branch tend to exhibit consistent expression changes.

### 3.7 Heterologous expression of TaNRAMP005 and TaNRAMP006 in yeast enhances cadmium tolerance

**Figure 10:**
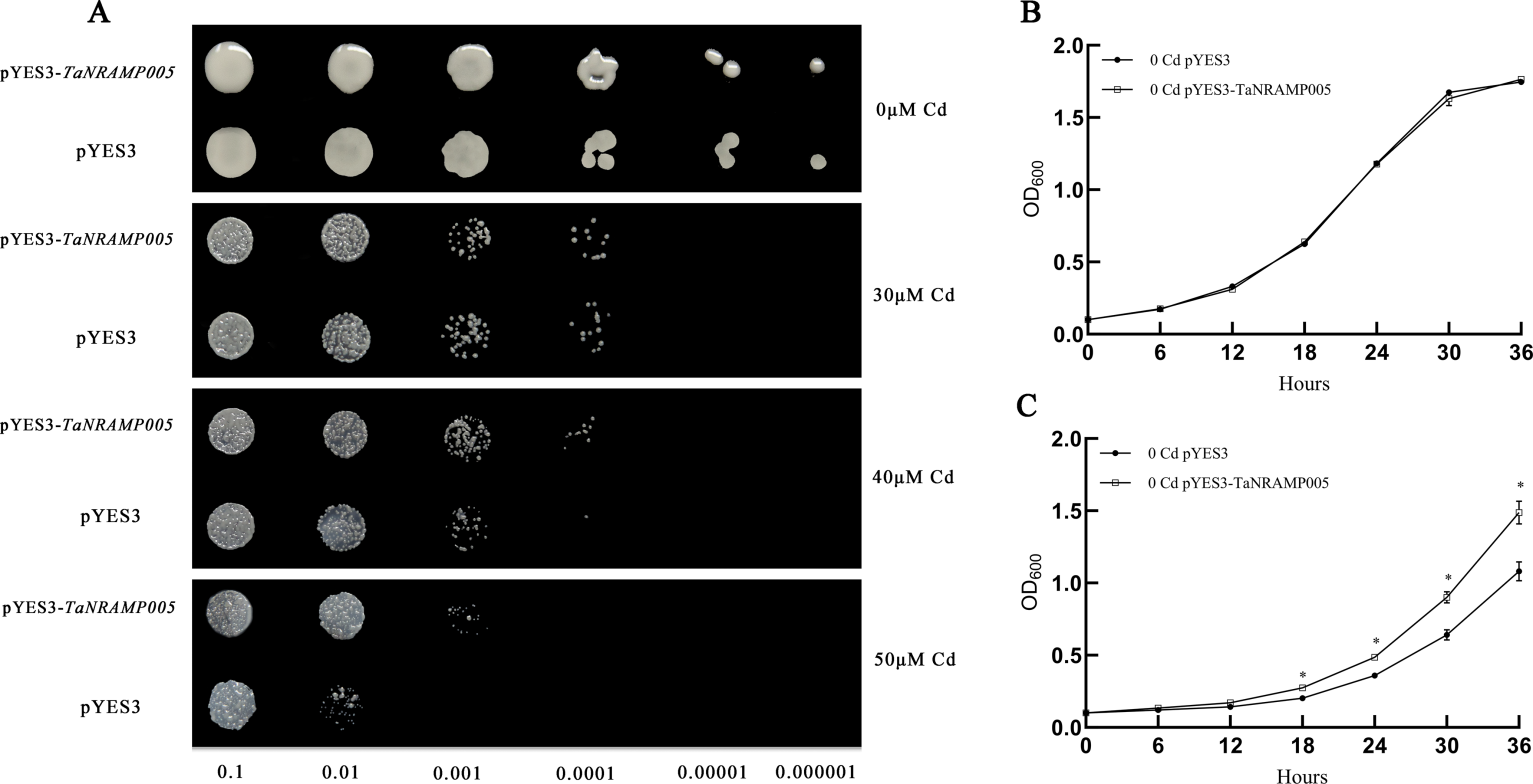
TaNRAMP005 enhances cadmium stress tolerance in yeast. **(A)** Effect of *TaNRAMP005* expression on cadmium tolerance. Yeast cells (Δycf1) carrying the empty vector pYES3 or *TaNRAMP005* were serially diluted from 10^-1^ to 10^-6^, spotted onto SG-Trp medium containing 0, 30, 40, or 50 μM cadmium, and incubated at 30 °C for 3 days. **(B)** Growth rate of yeast under cadmium-free conditions. Δycf1 strains harboring either the empty vector pYES3 or *TaNRAMP005* were inoculated into cadmium-free SG-Trp medium, and the optical density (OD_600_) of the culture was measured at predetermined time points. **(C)** Growth rate of yeast under cadmium stress. Δycf1 strains carrying the empty vector pYES3 or *TaNRAMP005* were inoculated into SG-Trp medium containing 30 μM cadmium, and the OD_600_ of the culture was measured at predetermined time points.

**Figure 11:**
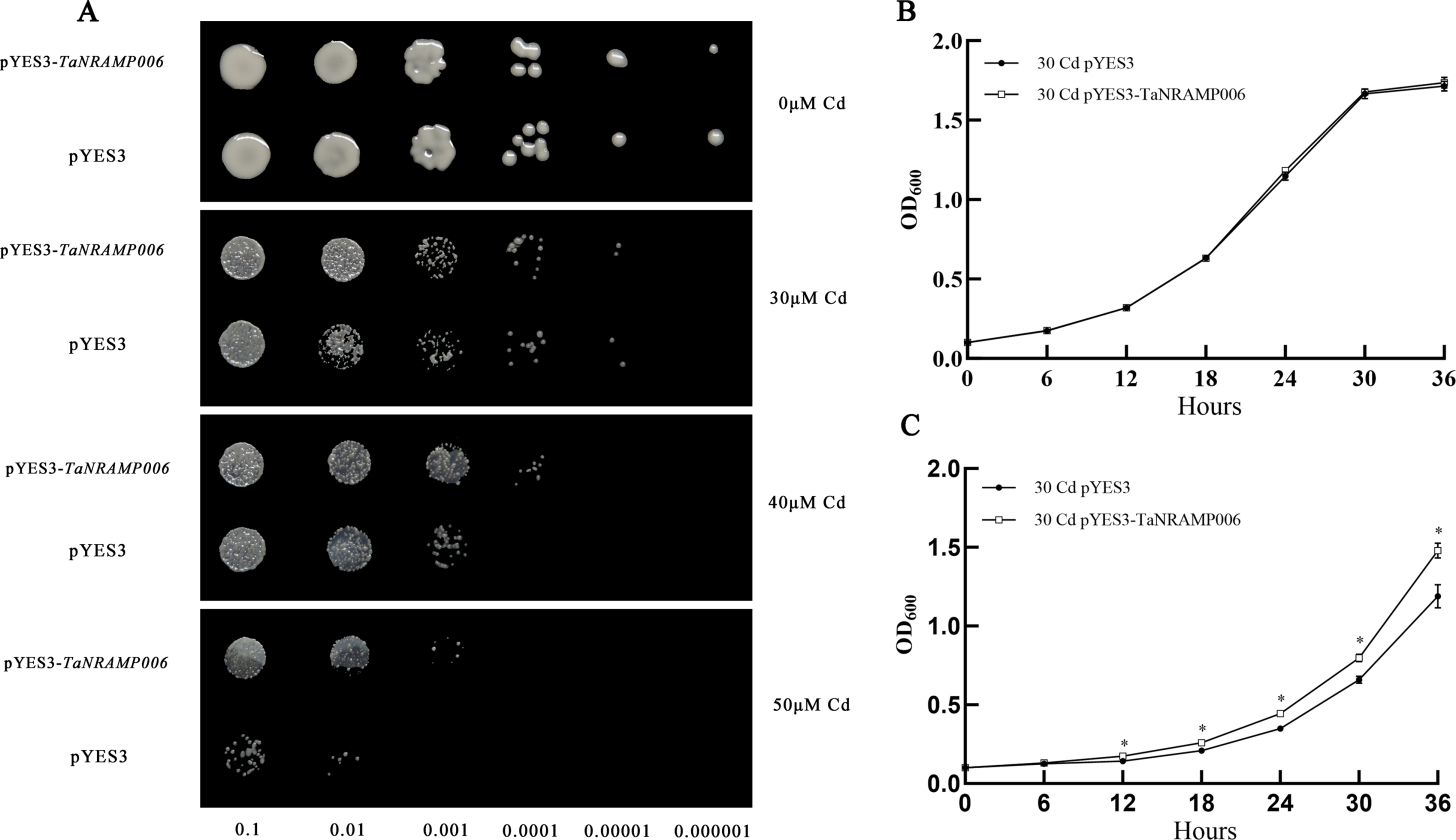
*TaNRAMP006* enhances tolerance to cadmium stress in yeast. **(A)** Effect of *TaNRAMP006* expression on cadmium tolerance. Yeast cells (Δycf1) carrying the empty vector pYES3 or *TaNRAMP006* were serially diluted from 10^-1^ to 10^-6^, spotted onto SG-Trp medium containing 0, 30, 40, or 50 μM cadmium, and incubated at 30 °C for 3 days. **(B)** Growth rate of yeast under cadmium-free conditions. Δycf1 strains harboring either the empty vector pYES3 or *TaNRAMP006* were inoculated into cadmium-free SG-Trp medium, and the optical density (OD_600_) of the culture was measured at predetermined time points. **(C)** Growth rate of yeast under cadmium stress. Δycf1 strains carrying the empty vector pYES3 or *TaNRAMP006* were inoculated into SG-Trp medium containing 30 μM cadmium, and the optical density (OD_600_) of the culture was measured at predetermined time points.

Under cadmium stress conditions, the genes *TaNRAMP005* and *TaNRAMP006* exhibited significantly upregulated expression. To verify their potential cadmium tolerance function, this study constructed recombinant expression vectors pYES3-*TaNRAMP005* and pYES3-*TaNRAMP006*, which were separately transformed into the cadmium-sensitive yeast strain Δycf1. Through plate resistance screening with different concentrations of CdCl_2_, it was found that under low cadmium concentrations (0 and 30 μM Cd), there was no significant difference in growth phenotype between the overexpression strains and the empty vector control strains. However, under 40 and 50 μM Cd cadmium concentration stress, the growth of yeast strains overexpressing *TaNRAMP005* and *TaNRAMP006* was significantly higher than that of the empty vector control group (**Figures 10A**, **11A**), indicating that both genes conferred enhanced cadmium tolerance to the yeast strains.

Liquid culture was performed in a constant temperature shaker at 30°C. The results showed that under blank treatment, the growth curves of the yeast strain carrying the empty pYES3 vector closely overlapped with those of strains harboring pYES3-*TaNRAMP005* or pYES3-*TaNRAMP006*, indicating that these two genes had no significant effect on yeast growth under normal conditions (**Figures 10B**, **11B**). Under 30 μM Cd stress, the OD_600_ values of strains carrying the above recombinant vectors were significantly higher than those of the empty vector control (**Figures 10C**, **11C**), consistent with the results from resistance plate screening. This further confirms that *TaNRAMP005* and *TaNRAMP006* are involved in the cadmium stress response.

## 4 Discuss

### 4.1 Identification and evolutionary relationships of the NRAMP gene family in wheat and its ancestral species

The NRAMP (Natural Resistance-Associated Macrophage Protein) family is an ancient membrane transporter family—first discovered and cloned in animals—that is also widely conserved in plants (Hall and Williams, 2003). It plays a critical role in regulating/maintaining metal ion homeostasis in plants and mitigating heavy metal ion stress (Yu et al., 2025). To date, NRAMP genes have been identified in model and crop plants such as rice, Arabidopsis, and soybean (Thomine et al., 2000; Inoue et al., 2024). In this study, we conducted a genome-wide survey of the NRAMP family in wheat and its ancestral species using ​protein sequence alignment​ and ​conserved domain comparison, identifying a total of 11 TuNRAMP genes (AA-genome type), 12 AeNRAMP genes (DD-genome type), 23 TtNRAMP genes (AABB-genome type), 22 TdNRAMP genes (AABB-genome type), 29 TsNRAMP genes (AABBDD-genome type), and 29 TaNRAMP genes (common wheat). Variations in gene copy number among ancestral species (AA, DD, AABB genomes) suggested dynamic gene loss/gain events during wheat polyploidization—most notably in the D subgenome: compared to its diploid ancestor *Aegilops tauschii*(12 AeNRAMP genes), common wheat retained only 8 TaNRAMP genes, indicate that selective gene loss events may have occurred during the evolutionary process. Subcellular localization prediction revealed that most NRAMP proteins are targeted to the plasma membrane, though a small subset was predicted to localize to the nucleus, vacuolar membrane, extracellular space, or chloroplast.

Phylogenetic tree analysis partitioned the NRAMP family into three subgroups (A, B, C), with Subgroup A containing the largest number of members. Calculations of the non-synonymous substitution rate (Ka), synonymous substitution rate (Ks), and divergence time showed that after gene duplication in the TaNRAMP lineage, outcomes included ​functional redundancy, ​functional differentiation, and ​conservation of ancestral functions. Synteny analysis between the wheat NRAMP family and its ancestral species (*Aegilops tauschii, Triticum turgidum, Triticum urartu, Triticum dicoccoides, and Triticum spelta*) revealed close evolutionary relationships: *Triticum spelta* shared 74 high-density synteny segments with common wheat, and average divergence times between *Triticum turgidum* and *Triticum spelta* were relatively short (13 mya and 23 mya, respectively). Moreover, the Ka/Ks ratios of most synteny gene pairs were <1, collectively indicating that these species retained highly similar NRAMP gene sequences with wheat during evolution—without significant functional divergence. Further analysis demonstrated that NRAMP genes were primarily constrained by ​purifying selection​ throughout evolution, ensuring the conservation of core functions like heavy metal transport. Additionally, gene duplication and species differentiation synergistically drove diversity in the NRAMP family: 29 duplication events within wheat, combined with differential duplication fragment numbers across ancestral species (e.g., 74 pairs with *Triticum spelta* vs. only 14 pairs with *Triticum urartu*), not only reflected significant expansion of the NRAMP family during genome duplication/polyploidization but also laid the groundwork for subsequent functional specialization.

### 4.2 Structural analysis, GO annotation, and promoter analysis of TaNRAMP genes

Structural analysis revealed significant ​conservation in motif composition​ among TaNRAMP members—particularly within phylogenetically clustered branches—alongside specific motif losses (e.g., motif_7 was absent in *TaNRAMP023/021*). Exon-intron architectures exhibited parallel evolutionary patterns, where closely related genes shared comparable intron-exon organizations. These findings indicate that TaNRAMP family members conserve core functional domains, while structural variations drive functional differentiation across the family.

GO functional annotation and promoter cis-element analysis provided crucial insights into the functional characteristics of TaNRAMP family members: both analyses indicate that these genes are likely involved in the transmembrane transport and homeostasis regulation of metal ions (including Fe, Mn, Zn, and Cd). Furthermore, the significant enrichment of various stress-responsive cis-acting elements (e.g., ABA, JA, SA, drought, and heavy metal response elements) in the promoter regions suggests that TaNRAMP transporters are highly likely to participate extensively in plant responses to abiotic stresses and hormonal signaling.

### 4.3 Expression patterns and potential functions of TaNRAMP

Previous studies have demonstrated that some NRAMP family members mediate Cd transport in specific plant tissues: *OsNRAMP1* (Takahashi et al., 2011) and *OsNRAMP5* (Tang et al., 2022) act complementarily in Cd uptake in rice, with *OsNRAMP5* reducing grain Cd accumulation. Arabidopsis *AtNRAMP6* (Cailliatte et al., 2009) and wheat *TaNRAMP5* (Yu et al., 2025) are also implicated in Cd absorption.

In our study, the *TaNRAMP005/012/016* and *TaNRAMP006/011/015* clade showed higher expression in roots than in other tissues and was ​upregulated under Cd stress, suggesting their potential involvement in regulating Cd tolerance mechanisms in the root system. Conversely, *TaNRAMP024/026/028* showed no Cd stress response, possibly complementing the *TaNRAMP005/012/016* clade. In our work, *TaNRAMP005/011/015* displayed higher root expression and were upregulated under Cd stress, implying they likely mediate root Cd tolerance. *TaNRAMP025/027/029* are upregulated under Cd stress, suggesting they may participate in Cd stress regulation.

*TaNRAMP002/003/004* are downregulated under Cd stress. We hypothesize *TaNRAMP002/003/004* may ​negatively regulate Cd stress responses. TaNRAMP018/021/023, which share expression patterns with *TaNRAMP002/003/004*, may also negatively modulate Cd tolerance, however, this hypothesis requires further experimental validation.

Transcriptome analysis results indicate that members of the TaNRAMP gene family exhibit varying degrees of response to cadmium (Cd) stress. Notably, under Cd stress treatment conditions, the expression levels of both *TaNRAMP005* and *TaNRAMP006* were significantly upregulated, suggesting that these two genes may actively participate in the plant’s regulatory response to Cd stress and possess potential cadmium tolerance functions.

### 4.4 TaNRAMP005/006 enhances yeast cadmium tolerance

Under cadmium (Cd) stress conditions, the significant upregulation of the *TaNRAMP005* and *TaNRAMP006* genes suggests that they may play crucial regulatory roles in wheat’s defense against cadmium stress. To further elucidate their molecular functions, this study employed a cadmium-sensitive yeast mutant (Δycf1) for heterologous expression validation. The results showed that compared to the empty vector control, yeast strains overexpressing either *TaNRAMP005* or *TaNRAMP006* exhibited no significant growth inhibition under low-concentration Cd treatment (0–40 μM). However, under high-concentration Cd stress (50 μM Cd), the transgenic strains demonstrated significantly enhanced cadmium tolerance. The findings from plate resistance screening were highly consistent with those from liquid culture growth curve analysis, confirming the biological function of these two genes in alleviating cadmium-induced cytotoxicity. Based on the integrated plasma membrane localization predictions from bioinformatics and yeast functional complementation evidence, *TaNRAMP005*​ and *TaNRAMP006​* are likely to respond to cadmium stress and function at the plasma membrane, thereby enhancing cellular tolerance and regulating cadmium resistance in wheat.

## 5 Conclusion

This study systematically identified the genome-wide distribution characteristics of the NRAMP (Natural Resistance-Associated Macrophage Protein) gene family in common wheat (*Triticum aestivum L.*) and its ancestral species. Based on phylogenetic analysis and genomic synteny mapping, the evolutionary trajectory and divergence patterns of the family were revealed. Further insights into the conservation of sequence features and functional diversification were obtained through analyses of gene domain composition and conserved motif characteristics. GO functional annotation and promoter cis-regulatory element analysis provided critical clues for deciphering the biological functions of family members and constructing transcriptional regulatory networks. Analysis of tissue-specific expression patterns and responses to cadmium (Cd) stress demonstrated that TaNRAMP family members exhibit tissue-specific expression, with significant differences in their response patterns to Cd stress. Notably, *TaNRAMP005* and *TaNRAMP006* were significantly upregulated under Cd stress. Yeast functional complementation assays further demonstrated that *TaNRAMP005* and *TaNRAMP006* significantly enhanced yeast tolerance to cadmium. Integrating the predicted plasma membrane localization features with the results of yeast complementation assays, we speculate that *TaNRAMP005* and *TaNRAMP006* are localized to the plasma membrane, respond to cadmium stress, and likely participate in the regulation of cadmium stress in wheat. These findings lay a theoretical foundation for future investigations into the functions of NRAMP genes and the molecular mechanisms underlying Cd tolerance in wheat.

## Data Availability

The datasets presented in this study can be found in online repositories. The names of the repository/repositories and accession number(s) can be found in the article/**Supplementary Material**.
